# Inter-population differences in acetabular senescence: relevance in age-at-death estimation

**DOI:** 10.1007/s00414-023-02954-x

**Published:** 2023-02-01

**Authors:** Marta San-Millán, Carme Rissech

**Affiliations:** 1 Medical Sciences Department, Clinical Anatomy, Embryology and Neuroscience Research Group (NEOMA), Faculty of Medicine, University of Girona, Carrer Emili Grahit, 77, 17071 Girona, Spain; 2 EUSES University School of Health and Sports, University of Girona, Carrer Francesc Macià, 65 17190, Salt (Girona), Spain; 3 Unit of Human Anatomy, Department of Basic Medical Sciences, Faculty of Medicine and Health Sciences, Rovira I Virgili University, Carrer de Sant Llorenç, 21, 43201 Reus (Tarragona), Spain

**Keywords:** Acetabulum, Age-at-death estimation, Aging, Accuracy

## Abstract

Since investigation of the timing of the skeletal traits among the acetabula of different populations is lacking, this study aims to evaluate the relevance of geographical origin in the acetabulum aging process and in the usability of the SanMillán-Rissech aging method. The acetabula of 826 European North Americans derived from the Bass Collection (USA) have been analyzed and compared with 611 Portuguese acetabula from the Luis Lopes Collection (Portugal) applying the most updated acetabular age estimation technique (2017). After evaluating and comparing the acetabular aging rates between both populations by Mann–Whitney *U* tests, the inaccuracy values (bias and absolute error) were analyzed and compared using population-specific reference samples and using references differing in geographical origin by Wilcoxon tests. In general terms, the North Americans age faster than the Portuguese, especially the females, reaching the consecutive acetabular stages at younger ages. Regarding the SanMillán-Rissech method accuracy, using population-specific reference samples produces, as a general rule, better outcomes. In addition, an exhaustive meta-analysis of inaccuracy values has demonstrated that this method provides better estimation values than pubic symphysis and auricular surfaces regardless of the geographic coherence of the reference sample. These inter-population skeletal differences are derived from different factors than age, highlighting the impact of both biological and social background on age estimation. A thorough analysis of the skeletal age-based timing becomes essential to understanding, deciphering and being able to minimize bias and potential inaccuracy or even counteract them when applying the age estimation methods to different populations.

## Introduction

While calculating physiological age in sub-adults is straightforward due to their predictable aging process, age-at-death estimation based on adult osteological material is one of the most difficult steps of an anthropological profile [1–5]. For this reason, a great deal of specific research is mainly focused on evaluating and improving the existing methods, as well as finding and investigating new age markers. Most of this investigation has concentrated on the *os coxae*, specifically on two of its joints: the pubic symphysis (e.g., [6–10]) and the auricular surface (e.g., [11, 12]). However, the third joint, the acetabulum, has not been recognized as a valid age marker until the current century. After some specific research in this line [13] and a preliminary combined approach of Rougé-Maillart et al. [14], Rissech et al. [15] published a novel age-at-death estimation technique based on morphological changes on the acetabulum exclusively for males. The usefulness of this methodology for aging estimation was also proved in different Western Europe populations with good accuracy results [16], which have been recently replicated in European Americans [17–19], Thai [20], and Colombians [21]. This aging technique is based on Bayesian inference, which has been extensively proved to offer good outcomes in anthropological disciplines [22–29]. This mathematical approach estimates the age-at-death of the target population based on prior probabilities from a reference sample [30].

Later, despite the Rissech method’s previously cited reasonably good results, and due in part to difficulties in the application, repeatability, and age correlation of the Rissech age-related variables of the acetabular fossa [31–33], San-Millán and coworkers revised the original technique, redefined these three specific variables, and evaluated the revised methodology in a documented Portuguese skeletal sample [34]. Unlike the original, this new approach was created based on a sex-pooled sample and was applicable to both biological sexes. Moreover, its results confirmed that it achieved high repeatability and easier application of the newly defined variables, with around 75% of the sample estimated with an absolute error lower than 10 years and a mean error of 7.28 years for males and 7.09 years for females [34]. Age-at-death estimation can now be calculated by both the original [15] and the revised version [34], using the renovated and user-friendly software linked to a freely IDADE2 web page (http://bass.uib.es/~jaume/IDADE2/https/index.html) fully described in Rissech et al. [30] (see the “Methods” section). The IDADE2 web page is written in the *R* statistical language and presented as a PHP web server and a web page user interface. This web page uses Bayesian inference to estimate age at death of unidentified individuals and/or samples. There, available reference samples are provided, offering the possibility to choose the most appropriate reference sample for a specific casework or research context. The freely provided reference datasets include samples from Spain [35], Portugal [34], the USA [18], and Colombia [21]. However, users can also apply their own reference sample data sets, making the web page adaptable to wider research questions.

For the last 20 years, much of the research regarding pelvic-based age-at-death estimation in adults has been based on testing and comparing the accuracy and reliability of the existing methodologies in geographically different documented skeletal collections. Despite that the majority of the research about population-based reliability has been performed in North America (the USA [e.g., [17, 36, 37] and Canada [31]) and Europe (England [33, 38, 39], Serbia [40], Poland [41], Italy [42], Greece [43–45], Spain [46, 47], France [48], and Portugal [49]), there have been some uncommon approaches based on samples from Asia (Thailand [20, 50, 51], Japan [52], China [53], and India [54]), South America (Chile [55] and Colombia [21, 56]), and Africa (South Africa [57, 58]).

Interestingly, most of the publications previously mentioned have included the acetabulum as a unique or part of the age markers within the key research question of the paper. Indeed, the acetabulum has become a focus of aging investigation within the last 10 years [18–20, 30–34, 39, 49, 57, 59–64], increasingly gaining popularity, reliability, and support from the scientific community linked to anthropological issues and lately appearing often along with other traditional age markers [17, 56, 65, 66]. Nevertheless, no international study has tested the SanMillán-Rissech method, the most up-to-date method of acetabular age estimation, outside Portugal [34] or the USA [18] thus far, except for a recent CT approach based on limited acetabular variables and, unlike the original, using principal component analysis and regression models which have achieved good accuracy results in Indian population [64].

Despite some inter-population differences in other pelvic age estimators’ timing that have been already reported in the literature (e.g., [67–69]), to the best of our knowledge, no other research has investigated the differences in the acetabular aging process rate between geographically distant populations. To bring knowledge to this poorly understood research line and as a continuation of our previous work on acetabular senescence [18, 34, 59, 70, 71], the present study aims to evaluate the relevance of geographical origin in the acetabulum aging process and in the usability of the SanMillán-Rissech aging method [34].

## Material and methods

### Material

The osteological material comes from two documented skeletal collections: the William Bass Donated Skeletal Collection [72, 73], housed in the Forensic Anthropology Center of University of Tennessee (Knoxville, TN, USA), and the Lisbon Collection, also called Luis Lopes Collection [74], housed at the Bocage Museum of the University of Lisbon (Lisbon, Portugal). The William Bass Collection is continually growing as a result of the establishment of a body donation program, and it now consists of over 1800 individuals, one of the largest collections of contemporary human skeletons in the USA. Dates of death range from 1977 and 2013. On the other hand, the Collection of Identified Human Skeletons, curated at the Bocage Museum (National Museum of Natural History, University of Lisbon, Portugal), is one of the largest and best preserved European anthropological collections. It originated from modern cemeteries, and, currently, it comprises 1692 identified skeletons, of which only 699 are currently documented and available for study. Dates of death range from 1880 to 1975.

From these two collections, males and females with completely fused acetabula were chosen for the analysis. From the Bass Collection, which is composed by individuals from different US populations (e.g., European, African, Asian), only European Americans were chosen to this study. This decision was made based on the very low sample size available for African and Asian individuals (54 and fewer than 10, respectively), together with the convenience for comparative purposes with the Portuguese sample. On the other side, all the individuals belonging to Luis Lopes Collection have European ancestry, so no restrictions were done in this sample. Specimens with evident pathologies affecting the acetabulum were not included. However, individuals with non-inflammatory osteoarthritis or diffuse idiopathic skeletal hyperostosis (DISH) were considered because both are indicators of aging [75, 76]. Thus, under these criteria, 826 North Americans (456 males and 370 females from 19 to 101 years of age) and 611 Portuguese individuals (294 males and 317 females from 15 to 98 years of age) were analyzed. Information regarding sex and age distribution along these samples is displayed in Table 1. From the whole sample, the left *os coxae* were assessed. The right side was evaluated when the left was damaged, pathological, or unavailable.

**Table 1:** Distribution of analyzed individuals by sex, age, and sample

	Portuguese		North American	
	Male	Female	Male	Female
< 20 years	12	13	1	0
20–29 years	24	25	9	2
30–39 years	24	14	40	14
40–49 years	48	18	74	38
50–59 years	57	47	84	75
60–69 years	48	45	101	98
70–79 years	40	85	89	78
80–89 years	41	60	51	48
≥ 90 years	0	10	7	17
Total	294	317	456	370
Reference	184	201	300	220
Test	110	116	156	150
Total	611		826	

### Acetabular age-at-death estimation

The seven acetabular variables used as age-related traits were substantially described, depicted, and evaluated by San-Millán et al. [34]. To evaluate the intra- and inter-observer error, weighted kappa statistic tests for ordinal data were performed [77–79]. The acetabular variables were evaluated by three different observers (the first author and two anthropology PhD students) using only the exhaustive descriptions and images provided by the original research [34]. Given that the first author of the current study is the person who developed this acetabular methodology revision [34], intra-observer error was carried out by her using the Lisbon collection [74]. However, given the impracticality for the two additional raters to analyze the Lisbon collection, inter-observer error was performed by using the Olmeda collection, an archaeological collection housed at the University of Barcelona (Barcelona, Spain, Medieval period). To evaluate intra-observer error of the seven acetabular variables, 60 left os coxae were chosen randomly (30 males and 30 females) from the Lisbon Collection and evaluated twice, 1 month apart, by the first author. On the other hand, to quantify inter-observer error, 37 left innominate bones from La Olmeda were examined by all three mentioned observers. In the latter case, only the three newly defined variables were evaluated [34] since the first four original variables of the Rissech’s method [15] have already demonstrated good levels of repeatability [16, 17, 33].

In the analyzed samples, every acetabulum was visually assessed by placing it into one of the described morphological states of each of the seven variables. Age-at-death estimates for every test specimen were calculated by entering acetabulum data in the IDADE2 web page ([30] http://bass.uib.es/~jaume/IDADE2/https/pages/benchmark.html*)*, based on frequencies of a reference sample and the Bayesian inference methodology used by Rissech et al. [15] and described in detail by Lucy et al. [22]. When applying the Bayesian inference here, the a priori* probability* of any 5-year age-at-death class was taken to be the fraction of individuals in the reference collection in that age-at-death class. It was assumed that each individual whose age-at-death was estimated is a sample of the population represented by the reference collection. An estimate of age-at-death takes the form of a probability distribution over 5-year wide age-at-death classes: 15–19, 20–24, etc. A single year estimate of age-at-death was calculated as the expected value of this distribution, attributing to each age class its central age. Complete, precise, and in-depth information regarding statistical underlying the acetabular methodology is supplied in Rissech et al. [30] and in the web page itself.

### Methods

The geographical origin of the reference sample has the potential to impact the resulting age estimates, since differing age estimates are given by the IDADE2 web page [30] depending on the available (or customized) reference sample/distributions chosen. To investigate this possibility, this study conducted different experiments (Table 2). Experiments 1 (age-at-death estimation of Portuguese based on Portuguese reference sample) and 2 (age-at-death estimation of European Americans based on European American reference sample) had already been performed and their results published [34, 18, respectively]. These data were taken as a baseline for comparison purposes. In order to evaluate the influence of the geographical origin of the reference sample on the aging outcomes, two more specific analyses were performed (Table 2): estimation of the Portuguese based on the European American reference sample (experiment 3) and age-at-death estimation of the European Americans based on the Portuguese reference sample (experiment 4). In addition, due to the existence of sex differences in the aging rate of the acetabulum aging process [33, 34, 70], males and females of each sample were analyzed separately with sex-specific reference samples in the four experiments performed.

**Table 2:** Different experiments performed in the current study according to sample size, geographical origin, and biological sex

Experiment	Reference sample	Test sample
1	Portuguese 184 males 201 females	Portuguese 110 males 116 females
2	European Americans 300 males 220 females	European Americans 156 males 150 females
3	European Americans 300 males 220 females	Portuguese 110 males 116 females
4	Portuguese 184 males 201 females	European Americans 156 males 150 females

To assess the age estimates produced by the four experiments, the bias and the absolute error between estimated age and chronological age were evaluated. Bias and absolute error are considered good indicators of a method’s inaccuracy [38]. Bias is the statistical measure that identifies the direction of the difference between the estimated and chronological ages [37, 42, 80], i.e., whether the age is over- (positive value) or underestimated (negative value). Bias was calculated as the average difference between estimated age and chronological age (∑(estimated age − chronological age)/*n*). On the other hand, absolute error is the statistical measure that evaluates the degree of the method’s inaccuracy. Absolute error was calculated as the average absolute difference between estimated age and chronological age (∑|estimated age − chronological age|/*n*). This parameter does not take into account the sign (positive or negative) of the difference between estimated age and chronological age [37, 42, 80], so it is independent of the inaccuracy direction.

#### Inter-population differences in the acetabular aging process

To evaluate the possible inter-population differences between Portuguese and North Americans in the rate of the aging process for the seven acetabular variables independently, following the same protocol as in the original paper [34], Mann–Whitney *U* tests were performed on the mean age of every variable stage. This analysis was performed separately in males and females. Since age estimation procedure is not required for this stage of the study, the complete samples were used to enlarge the sample size, without splitting it between *Test* and *Reference*, i.e., 456 North American males, 370 North American females, 294 Portuguese males, and 317 Portuguese females (Table 1). To complement these analyses and facilitate their visualization, box plots of known age reaching the different stages of each variable were explored visually. As usual, median, first and third quartiles, and maximum and minimum values have been considered for these graphics.

#### The relevance of population-specific reference samples in age-at-death estimation accuracy

First, the accuracy of the acetabular methodology was evaluated comparing the bias and absolute error mean values resulting from experiments 1, 2, 3, and 4 (Table 2). Experiments 1 and 2 used population-specific reference samples and experiments 3 and 4, *different from the test* population references. To enable comparisons, *Test* and *Reference* samples of both Portuguese and North Americans are always the same as specified in the methodology section (Table 1). Due to the fact that the same individuals (*Test* samples) were evaluated with different *Reference* samples, Wilcoxon tests to related samples were performed to compare the respective accuracies (bias and absolute error) in age estimation. In addition to the global sample, three wide age ranges were used: < 40 years, 40–64 years, and > 65 years. These specific age groups were chosen following the same protocol as San-Millán et al. [34] to ensure a reasonable sample size to statistical analyses, particularly in the youngest group.

Non-parametric testing was used throughout the study due to some small sample sizes, especially younger individuals, and because the normal distribution of some of the variables considered in the analysis cannot be assumed. All the statistical analyses were performed by SPSS 24.0 software, and 0.05 was accepted as the threshold to discriminate significant from non-significant results.

## Results

### Observer agreement

Intra-observer analyses of the variables indicated good levels of consistency, having values of weighted Kappa higher than 0.73 with *p* < 0.001 in each of the seven variables, suggesting substantial to perfect agreement [79]. Inter-observer analyses also showed substantial to perfect agreement between the three observers for the subset of variables tested. Values were higher than 0.67 with *p* < 0.001 in all cases [79]. According to Landis and Koch [79], a weighted kappa score of 0.61–0.79 (25% of the cases evaluated) denotes substantial agreement, while a score of 0.80–1.00 (75% of the cases evaluated) represents an almost perfect agreement. Because of the high level of consistency, the effects of possible observation error in the scoring process were considered negligible.

### Inter-population differences in the acetabular aging process

**Fig. 1:**
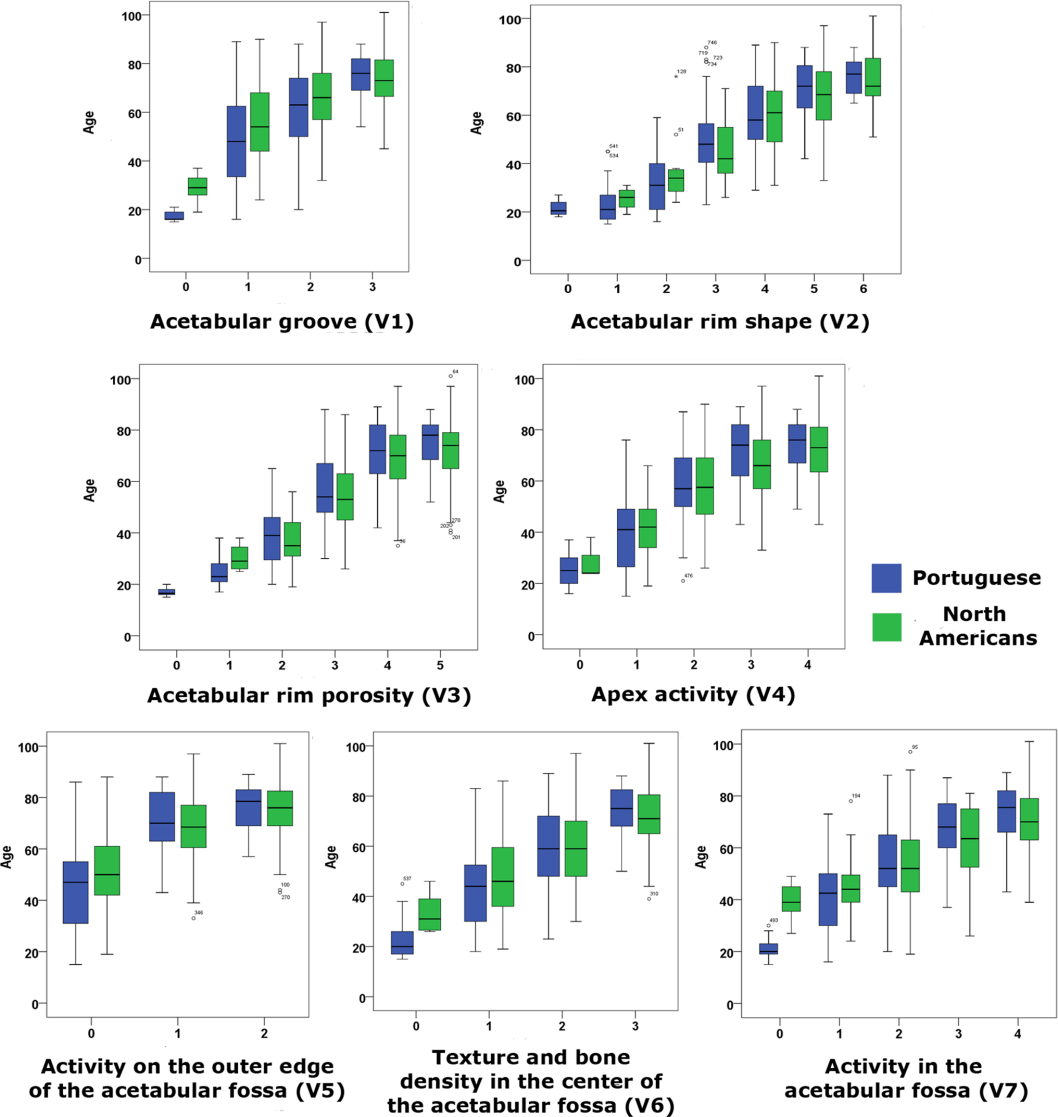
Box plots between age and each of the seven acetabular variables (V1–V7) to illustrate the relationship between the stage of each variable (horizontal axis) and age (vertical axis) comparing Portuguese (blue) and North American (green) males. The central line in the boxes indicates the median; the bottom and top of the box represent the first and third quartiles, respectively; and the ends of the whiskers correspond to the minimum and maximum values

**Fig. 2:**
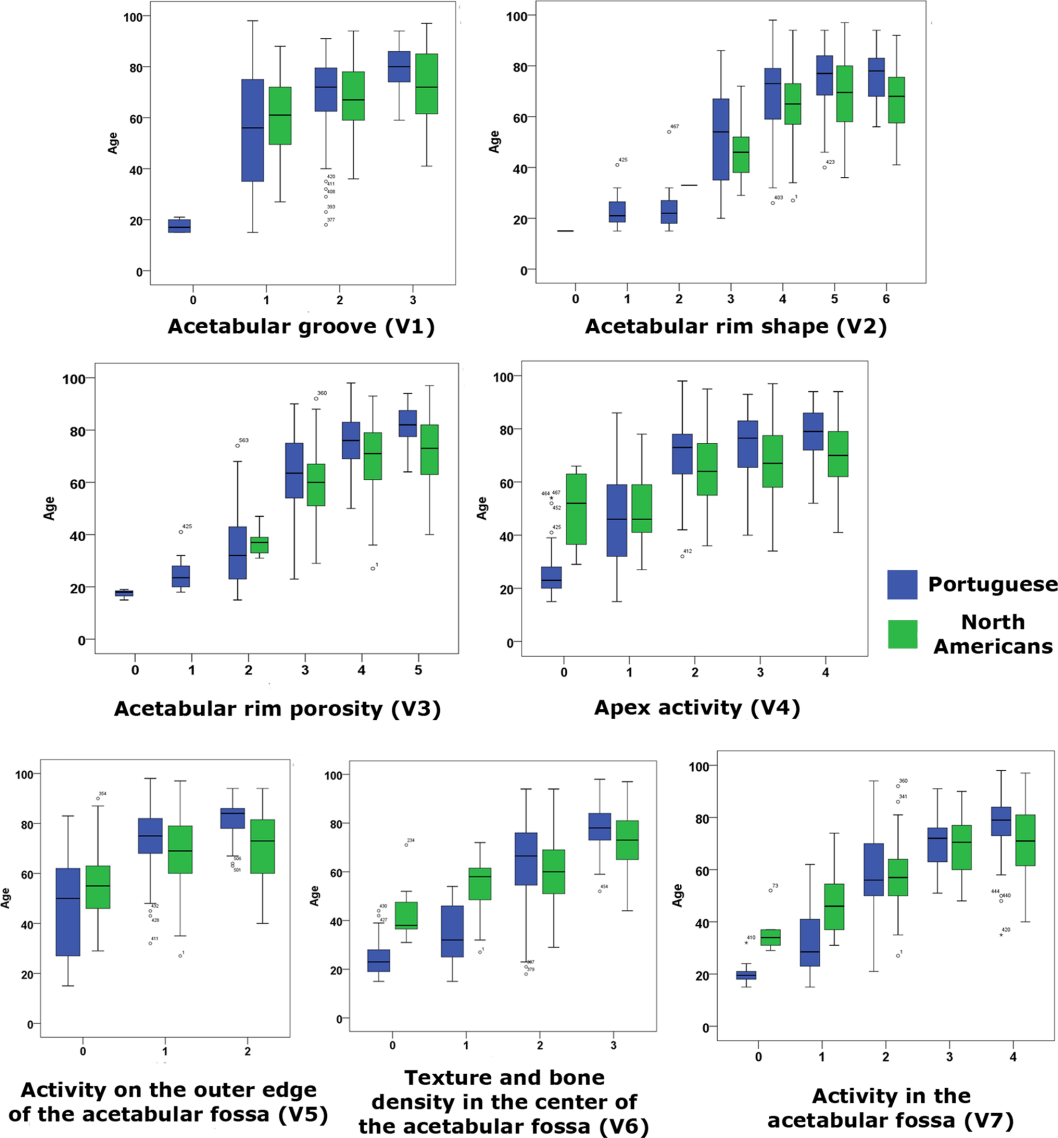
Box plots between age and each of the seven acetabular variables (V1–V7) to illustrate the relationship between the stage of each variable (horizontal axis) and age (vertical axis) comparing Portuguese (blue) and North American (green) females. The central line in the boxes indicates the median; the bottom and top of the box represent the first and third quartiles, respectively; and the ends of the whiskers correspond to the minimum and maximum values

To further investigation of acetabular aging rate between populations, male (Table 3; Fig. 1) and female (Table 4; Fig. 2) Portuguese and European American samples were evaluated, analyzed, and compared. It must be noted that, following the original methodology [34], mean averages are statistically compared in Tables 3 and 4, whereas median-based box plots were displayed in Figs. 1 and 2, so tables and figures are complementary, not entirely equivalent.

**Table 3:** Descriptive statistics of the mean chronological age of males for every stage of each variable, in Portuguese and North Americans separately. Right column displays results of the Mann–Whitney analyses that evaluate the inter-population differences in mean chronological age for every stage of the variables for each sample pair. Significant differences are marked in bold

Males		Portuguese			North Americans			Mann–Whitney *U*	*p*
Variables	Stage	*n*	Mean	SD	*n*	Mean	SD		
**1**	0	5	17.40	2.51	6	28.86	6.27	1.5	**0.013**
	1	131	48.04	19.16	250	55.90	15.68	12,425.5	**0.000**
	2	131	60.61	16.25	157	65.68	12.98	8622	**0.018**
	3	27	74.74	9.20	43	74.00	12.09	540	0.625
**2**	0	4	21.50	3.87	-	-	-	-	-
	1	20	24.20	9.20	4	25.50	5.00	29	0.393
	2	33	32.64	12.11	11	37.18	15.10	148	0.363
	3	39	50.67	16.13	49	44.73	12.18	746	0.078
	4	130	60.19	13.85	226	60.09	13.78	14,666.5	0.980
	5	52	70.67	11.40	134	67.05	13.92	2957	0.110
	6	16	75.94	7.15	32	74.16	11.90	239.5	0.718
**3**	0	10	17.00	1.56	-	-	-	-	-
	1	17	24.88	5.80	4	30.25	5.74	16	0.106
	2	51	37.84	12.01	30	36.73	9.17	723.5	0.685
	3	120	57.13	12.44	191	53.55	11.98	9728	**0.025**
	4	61	71.02	11.54	126	68.59	12.49	3402	0.204
	5	35	75.09	9.42	105	71.90	12.43	1527.5	0.135
**4**	0	17	24.71	6.25	3	28.67	8.08	18	0.426
	1	79	39.09	14.79	52	41.92	11.33	1836	0.305
	2	119	59.49	13.36	196	58.01	14.27	10,887.5	0.323
	3	45	71.40	12.93	114	66.04	12.94	1921.5	**0.014**
	4	34	74.65	9.56	91	71.19	13.26	1322.5	0.213
**5**	0	174	45.15	16.80	217	51.24	13.83	15,096.5	**0.001**
	1	102	69.58	12.21	196	67.85	12.65	9177.5	0.246
	2	18	76.94	8.63	43	74.98	12.41	345.5	0.511
**6**	0	26	22.58	7.51	11	33.55	7.79	38.5	**0.001**
	1	47	42.85	15.59	59	48.54	14.95	1085	0.055
	2	177	59.16	15.43	259	59.22	14.60	22,661	0.840
	3	44	74.20	10.05	127	71.43	11.49	2380	0.143
**7**	0	21	21.29	3.89	7	39.43	7.74	2.5	**0.000**
	1	34	40.91	15.36	35	44.43	11.31	516	0.343
	2	142	53.56	15.53	170	53.35	14.74	11,825	0.757
	3	27	67.67	12.89	44	62.30	13.61	462	0.118
	4	70	72.40	11.75	200	70.00	12.03	6037.5	0.087

**Table 4:** Descriptive statistics of the mean chronological age of females for every stage of each variable, in Portuguese and North Americans separately. Right column displays results of the Mann–Whitney analyses that evaluate the inter-population differences in mean chronological age for every stage of the variables. Significant differences are marked in bold

Females		Portuguese			North Americans			Mann–Whitney *U*	*p*
Variables	Stage	*n*	Mean	SD	*n*	Mean	SD		
Variable 1	0	4	17.50	3.00	-	-	-	-	-
	1	157	54.89	21.82	172	60.29	14.36	12,050	0.092
	2	115	69.09	14.76	146	67.86	12.43	7410	0.104
	3	41	79.51	9.28	52	72.60	14.93	784	**0.029**
Variable 2	0	1	15.00	-	-	-	-	-	-
	1	16	23.38	6.98	-	-	-	-	-
	2	17	23.88	9.17	1	33.00	-	-	**-**
	3	51	52.61	18.92	25	47.04	11.91	508	0.152
	4	158	69.05	14.00	200	64.78	13.24	12,505.5	**0.001**
	5	63	74.11	12.72	116	69.07	14.14	2886.5	**0.020**
	6	11	75.91	12.47	28	66.96	13.11	96	0.070
Variable 3	0	4	17.50	1.73	-	-	-	-	-
	1	14	25.07	6.71	-	-	-	-	-
	2	42	33.00	14.20	9	37.44	5.43	128.5	0.135
	3	128	63.49	13.69	159	59.64	12.15	8290.5	**0.007**
	4	85	75.02	9.90	129	69.39	12.69	4071.5	**0.001**
	5	44	81.39	7.38	73	72.34	14.46	995.5	**0.001**
Variable 4	0	29	26.76	11.06	4	49.75	16.66	9.5	**0.007**
	1	69	46.59	19.07	31	48.84	12.76	983.5	0.521
	2	130	70.62	11.58	171	64.73	13.70	8149.5	**0.000**
	3	52	73.50	12.84	87	67.36	13.44	1618.5	**0.005**
	4	37	78.35	10.72	77	70.26	12.67	881	**0.001**
Variable 5	0	134	46.54	20.03	111	55.29	12.66	5553	**0.001**
	1	158	73.56	11.09	211	68.78	12.93	12,876	**0.000**
	2	25	81.28	8.60	48	70.90	13.96	324	**0.001**
Variable 6	0	33	24.61	7.67	7	44.00	13.61	17	**0.000**
	1	21	34.76	12.42	19	54.63	12.02	52.5	**0.000**
	2	168	65.14	15.05	189	60.48	13.62	12,461.5	**0.000**
	3	95	77.97	8.27	155	72.74	11.44	5284	**0.000**
Variable 7	0	14	20.07	4.16	6	36.17	8.33	2.5	**0.001**
	1	38	32.21	12.74	15	46.67	12.02	112	**0.001**
	2	90	57.28	14.99	109	56.85	11.23	4712.5	0.634
	3	53	70.09	9.47	44	68.89	11.37	1110.5	0.687
	4	122	78.01	9.46	196	70.95	12.53	7721	**0.000**

Because of the low number of young individuals in the North American collection, some of the results in the first stages should be interpreted carefully, even being statistically significant (variables 1, 5, 6, and 7 in males and variables 4, 5, 6, and 7 in females). Thus, the mean ages of Americans could be higher in these initial stages due to the sample bias (Table 1) and not because of biological causes. In fact, when enough sample size was available, the opposite pattern to the generally found in this study (see below) was observed, i.e., significantly higher mean ages were found in the European American sample compared with the Portuguese in the first stage of the variables in both sexes, with the exception of variable 4 in males (Table 3; Fig. 1). In this specific case, the trend was identical but the differences were not statistically significant.

Thus, general analyses of Tables 3 and 4 suggest that, considering the 0 and, possibly the 1 stages, as a not-aged mature morphological pattern, European Americans reach the consecutive aging stages at younger mean ages than Portuguese, meaning that the former age faster than the latter. In the case of males (Table 3; Fig. 1), however, these differences are only significant in stages 3 of variables 3 and 4, while the significant results appeared throughout the female analyses (Table 4). In most of the rest of the stages, specifically the last two or three stages of every variable, European American females reached the progressive age-related phases significantly earlier than Portuguese (Table 4; Fig. 2). This means that the acetabular aging rate of European American females is, in general terms, significantly higher compared with the analyzed Portuguese females, i.e., European American females ages faster than Portuguese females. It seems that the inter-population aging patterns and rates are more populationally disparate in females than in males. Unlikely and as a unique exception, regarding variable 1 (acetabular groove), the opposite pattern was found in males, being the Portuguese who reached all the stages at a significantly early mean age (stages 1 and 2 in Table 3; Fig. 1).

### The relevance of population-specific reference samples in age-at-death estimation accuracy

Aimed to investigate the importance of the populational origin of the reference sample in the age-at-death estimation based on the acetabulum, this study compared the accuracy of this method to the same test samples, combining population-specific reference samples (experiments 1 and 2) and non-population-specific ones (experiments 3 and 4). In the application to the Portuguese sample (experiments 1 vs 4) and with respect to bias, significantly better results were provided when using the same population as a reference on the whole sample in both sexes (Table 5, top), with significantly more underestimation when distant populations were used as a reference. Similar results were found in the oldest age group, but in 40–64 years old males, the results were also significantly different but in different direction; using the same reference population slightly overestimated the results, and using a diverse reference resulted in slight underestimation. The same results were reported in middle-aged females, but the results were not significantly different. For the younger group, overestimation was the rule, but the differences were not significant in any sex. On the other hand, and in relation to absolute error (Table 5, bottom), better results were achieved in the Portuguese sample when the Portuguese instead of North American reference collection was applied to both males and females. However, in this case and unlike the North American sample (see below), the results were not statistically significant. In the different age groups, using same population as reference gave significantly better accuracies in the oldest age group in both sexes. However, better accuracy values were reported when non-specific-population reference samples were used in the rest of the age groups (except for the youngest female group) even though only in the middle-aged female group does the differences were statistically significant.

**Table 5:** Mean differences in bias and absolute error between (1) same Portuguese individuals estimated by using either the Portuguese reference sample (experiment 1) or the European American reference sample (experiment 4) and between (2) same European American individuals estimated by using either the European American reference sample (experiment 2) or the Portuguese reference sample (experiment 3) in both sexes separately. Due to exactly the same individuals were evaluated in the comparison, Wilcoxon test to related samples were performed in the whole sample and in every age group independently. Significant differences are marked in bold

Bias										
	Males					Females				
	Experiment 1 (Portugal/Portugal)	Experiment 4 (Portugal/North America)	*n*	Wilcoxon	*p*	Experiment 1 (Portugal/Portugal)	Experiment 4 (Portugal/North America)	*n*	Wilcoxon	*p*
15–39 years	4.23	3.69	16	− 0.569	0.569	3.90	11.54	5	− 1.753	0.080
40–64 years	2.29	− 1.76	46	− 4.901	**0.000**	2.78	− 0.25	30	− 1.749	0.080
> 65 years	− 4.61	− 7.59	41	− 3.240	**0.001**	− 2.31	− 8.25	67	− 3.995	**0.000**
Global	− 0.16	− 3.23	103	− 5.440	**0.000**	− 0.51	− 4.93	102	− 3.963	**0.000**
	Experiment 2 (North America /North America)	Experiment 3 (North America/Portugal)	*n*	Wilcoxon	*p*	Experiment 2 (North America/North America)	Experiment 3 (North America/Portugal)	*n*	Wilcoxon	*p*
15–39 years	5.41	9.95	15	− 2.789	**0.005**	8.63	8.85	4	−	−
40–64 years	2.65	7.63	73	− 7.134	**0.000**	6.97	12.48	64	− 5.136	**0.000**
> 65 years	− 4.77	− 0.53	63	− 5.963	**0.000**	− 3.82	0.89	77	− 6.297	**0.000**
Global	− 0.17	4.46	151	− 9.729	**0.000**	1.28	6.22	145	− 7.955	**0.000**
Absolute error										
	Males					Females				
	Experiment 1 (Portugal/Portugal)	Experiment 4 (Portugal/North America)	*n*	Wilcoxon	*p*	Experiment 1 (Portugal/Portugal)	Experiment 4 (Portugal/North America)	*n*	Wilcoxon	*p*
15–39 years	6.86	5.06	16	− 1.916	0.055	8.66	11.54	5	−	−
40–64 years	6.78	6.25	46	− 0.344	0.731	9.46	7.49	30	− 2.037	**0.042**
> 65 years	8.67	11.32	41	− 2.709	**0.007**	6.54	9.95	67	− 2.936	**0.003**
Global	7.54	8.08	103	− 1.035	0.301	7.50	9.31	102	− 1.567	0.117
	Experiment 2 (North America/North America)	Experiment 3 (North America/Portugal)	*n*	Wilcoxon	*p*	Experiment 2 (North America/North America)	Experiment 3 (North America/Portugal)	*n*	Wilcoxon	*p*
15–39 years	5.95	10.62	15	− 3.415	**0.001**	8.63	8.85	4	−	−
40–64 years	7.46	9.69	73	− 3.953	**0.000**	10.35	14.01	64	− 3.932	**0.000**
> 65 years	7.45	6.16	63	− 2.306	**0.021**	9.07	7.79	77	− 1.397	0.163
Global	7.30	8.31	151	− 2.726	**0.006**	9.62	10.57	145	− 2.059	**0.040**

In the case of North Americans, the outcomes generated were quite different and with almost all the analysis found to be statistically significant. Table 5 shows that mean bias significantly differed between both analyses (experiment 2 vs. 3), with significantly better results by using a reference sample of the same population as the target one, except for the oldest group of both sexes, where the opposite pattern was observed. In this specific oldest age interval, using the Portuguese reference sample instead of the American one resulted in a bias close to zero, while to use same target and reference population underestimated the age-at-death on average. Thus, when the global results in both North Americans and Portuguese are considered, using population-specific reference sample yields results similar to zero, while using non-specific-population as a reference in the age estimation yields opposite results: overestimation in North Americans using the Portuguese reference and underestimation in Portuguese when using the North American reference. Those results are consistent along both sexes. Similar results were found with regard to absolute error in European Americans (Table 5, bottom). The age-at-death was significantly more accurate when population-specific reference sample were used to the analyses in all cases, including both the overall sample and all specific age intervals except the oldest one. In this latter case, European Americans older than 65 years of age were estimated significantly more precisely using the Portuguese reference sample instead of the American one, though only significantly in males.

In addition to the previous outcomes, a complementary and noteworthy result should be highlighted. Together with the acetabular-based estimation inaccuracy mean values previously mentioned, the implementation of the method is equally essential. Thus, Table 6 shows how the applicability of the SanMillán-Rissech method decreases substantially when non-specific-population samples were used as references (mean value 1 vs. 6.75); in essence, the IDADE2 web page [30] sent back “no estimation” results in a considerably higher number of cases.

**Table 6:** Number of not estimated individuals through the IDADE2 software taking into consideration the biological sex and the population origin of both the test and reference samples

Test sample	Reference sample	Number of individuals not estimated by IDADE2 software	Mean
Female Portuguese	Female Portuguese	0	1
Male Portuguese	Male Portuguese	1	
Female European American	Female European American	1	
Male European American	Male European American	2	
Female Portuguese	Female European American	14	6.75
Male Portuguese	Male European American	6	
Female European American	Female Portuguese	4	
Male European American	Male Portuguese	3	

## Discussion

### Inter-population differences

The present results have proved that significant differences do exist in the acetabular aging rates between geographically distinct samples with differing population histories (Tables 3 and 4). North Americans, both males and females, age faster than Portuguese, reaching higher acetabular stages earlier and showing more skeletal aging modifications at younger ages, comparatively. This finding is essential because these different aging rates determine the subsequent accuracy results (Table 5). In fact, a general underestimation of age occurred when Portuguese individuals were estimated based on the North American reference sample, because the latter age faster, arriving earlier at each age-progressive acetabular state and thus biasing the results. On the contrary, overestimations generally resulted when North Americans were estimated based on the Portuguese reference sample, since Portuguese age slower, reaching the same skeletal age later in time. Thus, investigating such differences in aging processes should be the first step in age-at-death estimation for every age marker, because this can determine the potential inaccuracy and bias of the methodology in different geographical contexts.

It seems that references from the same population, as close as possible in spatial and temporal background, or perhaps from populations with similar aging trends or rhythms, should offer the best outcomes for anthropologists [81]. In fact, some authors have already reported inter-population differences in the aging skeletal rates for other age indicators. Sinha and Gupta [67] found significant variation in the timing of pubic-based changes between North American and Indian samples. Hoppa [69] also observed differences in the age-progressive pubic changes between females from the USA and England. Also, Kimmerle et al. [82] reported aging differences between females from the USA and Balkans based on the pubis morphological changes. In this line, Schmitt [50] concluded that Suchey-Brooks and Lovejoy methods were not applicable to Thai samples. For these results among others, the utilization of population-specific reference was advised when possible [68].

Contrary to this push for population specificity, other researchers are attempting to amass large, diverse, global samples that are universally applicable [e.g., 83]. While a different means to the end of accurate age estimation, these large reference samples are specifically designed to account for population variation in rates of aging and have the advantage of being applicable even when an individual’s population of origin is unknown.

Some primary unexpected results were found in this study. For example, the bias was significantly reduced (closer to zero) when Portuguese were placed as reference instead of North Americans to test North Americans older than 65 years old of both sexes (Table 5, top). Likewise, the same pattern occurred in the middle-aged Portuguese males, displaying significantly better results when a non-population-specific reference sample was used (Table 5, top). It is interesting to emphasize that these counteracted outcomes occurred not by chance just earlier in life in Portuguese (when overestimation is the rule) and only later in life in North Americans (when underestimation is the most frequent pattern), specifically due to the different aging velocity between these both populations. Thus, it seems intuitive that knowing in advance the acetabular behavior in different populations would be a good opportunity to counteract the resulting bias using references that compensate any unbalances in the right directions.

In the same line, better absolute results when population-specific reference samples were used were the more expected outcomes. However, in some cases, using distant reference samples resulted in better accuracies in the present study (Table 5, bottom). Again, these unexpected results are understandable when current inter-population differences previously reported in the skeletal aging process are taken into consideration. Thus, due to European Americans aging faster in terms of skeletal acetabular traits, they take advantage of using Portuguese as reference since they reach the acetabular consecutive stages later. In this way, it is significantly compensated in the oldest age group. In the case of the Portuguese, the opposite happened: The compensation occurred in the middle and youngest age groups. These results make clear that the focus of our age-estimation studies needs to be changed. Instead of researchers simply testing the different methodologies in different populations across the globe, the emphasis should be placed first on the aging process itself. In essence, the answers are in the process (skeletal aging) more than in the results (accuracy method). Importantly, these answers could allow anthropologists to adjust the outcomes choosing the more convenient reference sample when available. Thus, Mays [84] has already recommended that rather than standardizing the different techniques, aging methodologies and standards need to be selected according to the appropriateness for a particular material under study.

### Understanding population differences: possible explanations for differing rates of aging

The inter-population differences exist, but the factors which potentially can cause the different rates are poorly understood in age-at-death estimation, since comprehending the physiological process of skeletal aging is a complex and challenging task. Added to all of this inter-population information, intra-population differences could be relevant, not only regarding the methodologies’ accuracy, but also about differences in metamorphic/aging rates attributable to diverse genetic background, microevolutionary history, eating habits, lifestyle, socio-economic status, workload, environmental factors, and a huge range of elements that would be taken into the aging equation. In fact, some investigations have concluded that just less than 50% of the variability of pelvic age indicators is associated with chronological age [19, 84–86]. As Mays [84] reviewed thoroughly, factors other than age that were responsible for variation in the aging process in the pubic symphysis, auricular surface, sternal rib end and cranial suture closure included parity, hormones, joint stress, joint mobility or laxity, physical activity, genetic factors, obesity, vitamin deficiency, energetic status, biomechanical forces, and lean muscle.

Despite limited research published in the previous century [87], it has not been until recently that many researchers have tried to deeply understand the aging process itself, its relationship with different linked factors others than age, and how those may be modifying the aging patterns and biasing the estimation. They have addressed the role of osteoarthritis [61, 65], osteoporosis [66], BMI and obesity [19, 88–90], occupation [33, 91], tendency to bone growth [92], biological sex [33, 34, 70], and embodied experiences of social inequity [93] in the progressive changes of pelvic-based age markers and their implications in the age-at-death estimation procedures.

While obesity seems to significantly affect age-related changes in the auricular surface in the USA [89], the pubic symphysis may be a more reliable indicator of age in obese individuals [89], and the acetabulum has been proved to be resistant to BMI and obesity status [19] in the same geographical area. In this line, Merritt [88, 90] reported how body mass and stature can influence accuracy values in the pubic symphysis and auricular surface, globally under-aging subjects with light body mass and short-stature and over-aging heavy body mass and tall-stature individuals. However, Merrit [88] reported US individuals with low body size being who attain a given age-phase earlier in life. According to these data, overweight, obesity, nutrition, and lifestyle could be behind the existent differences in the acetabular aging rhythm between North Americans and Portuguese.

Despite Buk et al. [81] reporting that biological sex is not important in age classification regarding pubic symphysis and auricular surface methods, Mays ([33], English sample), San-Millán ([70], Portuguese sample), and San-Millán et al. ([34], Portuguese sample) demonstrated that the biological sex does play an important role in acetabulum topic because, even following a similar acetabular aging pattern, males age faster than females. Moreover, relationships between occupation and age indicators have not been supported in the pubic symphysis in a male Portuguese sample [91] or in the acetabulum of a male and female US sample [19]. However, in the acetabulum of eighteenth to nineteenth century English individuals, contrary to original hypotheses, Mays [33] found that skeletal individuals who had undertaken nonmanual professions showed greater acetabular scores-for-age than those in manual trades. This suggests that physical inactivity may be driving acetabular aging, a force which might also be at play in the relatively physically inactive modern US sample examined in the current study. Interconnections between osteoarthritis and age-at-death estimation have also been important to consider [65], helping in refining the estimated ranges and giving remarkable information in fragmentary contexts [61]. Besides, Mays [33] did not find any association between acetabulum aging process and bone generation (DISH), while Rissech et al. [66] stated that osteoporosis/bone loss has not significant influence neither in the pubic symphysis, nor in the auricular surface or in the acetabulum as age indicators. Thus, it is vital not only to assess and enhance the existing age-estimation methods, but also, and not less essential, to comprehend the social, cultural, and biological factors underlying the age-related anatomical process. Due to the scarcity of specific information regarding the factors other than age that modify the progressive pelvis changes used for age-at-death estimation, particularly in the acetabulum, further research in this line is completely necessary.

Since North America and Europe have markedly different historical and legal background, their documented collections could have potential different sources: body donation programs, medical examiner or forensic cases, autopsies, dissection cadavers, archaeological sites, and/or modern cemeteries. Consequently, they can vary in the cultural, socioeconomical, and biological status of their subjects [94]. The economic and personal profits that body donations programs can provide to the individuals and families may bias the collection’s characteristics. Body donation programs in the USA, for example, could receive subjects with different socioeconomical status and biological background than equivalent European programs [95]. In addition, secular trends are changing the donor standards over the time, distancing progressively from the “older than 65 years old while male” reported in the past literature [96, 97]. The biological and social characteristics of the sample may also differ from those coming from standard cemeteries. Also, the fact that many of the subjects, especially from cadaver dissections from nineteenth to twentieth centuries, are unclaimed human remains can also influence in their life context (e.g., impoverishment, drug consumption, homeless conditions, unhealthy habits, unsupportive family, uprooting) and, consequently, in age markers’ morphological progression. Thus, both demographic constitution and origin of the documented collections could be influencing the comparatively different results in diverse populations, including some bias that should be considered for further investigation. As mentioned in the materials section, while the North American sample was derived from twentieth to twenty-first centuries (dates of death from 1977 to 2013), the Portuguese sample was constituted during the nineteenth and twentieth centuries (dates of death from 1880 to 1975). However, according to Cardoso [74], the impact of the temporal divergency should not be such biasing since, despite 71.1% of the individuals of the documented available specimens from Lisbon (*n* = 699) were born during the nineteenth century, just 5 passed away during this century (0.71%). In fact, this 5 could not have been included in this study because only 611 out of the 699 individuals meet the inclusion criteria. This would suggest that most of the aging acetabular process took place in the twentieth century in both populations, effectively rendering the samples comparable and justifying the present results.

Nevertheless, this demographic and bio-social variability may also potentially be considered to understand the present findings. Some acetabular variables have recently been proposed as sensitive to lived experiences of chronic stress in modern US samples [93]; changes in social conditions from the nineteenth through twenty-first centuries, and the accompanying secular skeletal changes, may also play a role in the differing rates of aging observed in this study. If so, it is interesting, and perhaps unexpected, that individuals in the more recent North American sample aged faster than individuals in the historic Portuguese sample, suggesting that lived experiences of social inequity may be contributing to premature aging even in this modern, highly industrialized Western nation.

Regardless of the composition, origin or differences between reference collections, the most meaningful result of this study is that acetabulum-based age estimation garners better results in comparison to other routinely studied pelvic age markers, even when employing non-population specific references (see below).

### Meta-analysis of pelvic age markers

Finally, to be able to make comparisons regarding of methodologies’ imprecision, deep research of publications including a mean absolute error as an inaccuracy value has been performed for pelvic age markers and corresponding techniques: acetabulum (Table 7), pubic symphysis (Table 8), and auricular surface (Table 9). This meta-analysis demonstrates that acetabular traits described by Rissech et al. [15] originally and revised and complemented by San-Millán et al. [34] work properly enough across the world with similar inaccuracy mean values in different locations worldwide. In fact, mean errors are between 7.09 and 9.65 years when population-specific reference samples were used while barely higher than 10.5 years were reported using non-specific-population reference samples, including estimating ages of different ethnicities. Thus, the maximum value was reported when African Americans were estimated using European Americans as a reference (see Table 7; [71]). It needs to be acknowledged that the methodology proposed by Calce [32], even examining acetabular-based traits, does not seem to achieve such good results (Table 7). Thus, except for Calce’s method results, the acetabular-based committed mean errors in the age-at-death estimation are, as a global data and independently of using population-specific references, lower than the results of the application of methodologies using age-related features of the pubic symphysis (Table 8) and the auricular surface (Table 9) in diverse populations. The acetabulum has been proved to be a more accurate age marker, even when non-population-specific reference samples were used in comparison with other pelvic-based methodologies. Despite further research is always welcome, the current results and the presented meta-analysis should be enough evidence to take the acetabulum seriously in legal medicine, bioarcheology, and forensic anthropology contexts. In this line, acetabulum should the chosen as a priority over other pelvic methods, specifically over the popular pubic symphysis. In addition, acetabulum can offer a better preservation than pubic symphysis and auricular surface [98], which is an important issue to take into consideration in challenging conservation cases.

**Table 7:** Comparative review of the reliability (inaccuracy based on the mean absolute difference between the chronological and estimated ages-at-death) of different methodologies based on the age-related traits of the acetabulum. Regarding North American samples, (E) corresponds to European Americans, while (A) are formed by African Americans

Anatomical element	Age-at-death method	Reference sample geographical origin	Test sample geographical origin	Mean male inaccuracy	Mean female inaccuracy	Mean sexes-combined inaccuracy	References (chronologically)
	San-Millán et al., 2017 [34]	Portugal	Portugal	7.28	7.09	-	[34]
		USA (E)	USA (E)	7.19	9.65	-	[18]
		USA (E)	USA (A)	9.16	10.63	-	[71]
		USA (E)	Portugal	8.05	9.31	-	**Current study**
		Portugal	USA (E)	8.27	10.56	-	**Current study**
	Rissech et al., 2006 [15]	Spain	Spain	-	-	8.53	[35]
		Canada	Canada	-	-	8.00	[31]
		USA (E)	USA (E)	-	-	8.61	[17]
		USA (E)	USA (E)	8.14	10.53	-	[18]
		Thailand	Thailand	-	-	8.00	[20]
		Colombia	Colombia	9.44	10.63	-	[21]
	Calce, 2012 [32]	USA (E)	USA (E)	12.8	13.78	13.18	[17]

**Table 8:** Comparative review of the reliability (inaccuracy based on the mean absolute difference between the chronological and estimated ages-at-death) of different methodologies based on the age-related traits of the pubic symphysis. Regarding North American samples, (E) corresponds to European Americans

Anatomical element	Age-at-death estimation method	Test sample geographical origin	Mean male inaccuracy	Mean female inaccuracy	Mean sexes-combined inaccuracy	References (chronologically)
Pubic symphysis	Brooks and Suchey, 1990 [10]	Thailand	14.2	16.4	-	[50]
		Italy	13.6	13.8	-	[42]
		USA (E)	-	-	10.5	[37]
		USA (E)	20.3	17.57	19.27	[17]
		Spain	-	-	13.98	[35]
		Spain	12.87	16.04	-	[47]
		Thailand	9.2	12.5	-	[51]
		Colombia	-	-	12.49	[56]
		Greece	10.7	12.1	-	[45]
		Colombia	10.29	9.05	-	[99]

**Table 9:** Comparative review of the reliability (inaccuracy based on the mean absolute difference between the chronological and estimated ages-at-death) of different methodologies based on the age-related traits of the auricular surface. Regarding North American samples, (E) corresponds to European Americans while any indication together with “USA” means that different ethnicities were included in the study

Anatomical element	Age-at-death estimation method	Test sample geographical origin	Mean male inaccuracy	Mean female inaccuracy	Mean sexes-combined inaccuracy	References (chronologically)
Auricular surface	Lovejoy et al., 1985 [11]	USA	-	-	7.55	[11]
		Thailand	13.8	18.2	-	[50]
		USA	-	-	11.6	[100]
		USA (E)	-	-	11.6	[37]
		Spain	-	-	13.88	[35]
		Italy	11.4	12.8	-	[42]
		Colombia	-	-	9.77	[56]
		Greece	9.0	8.9	-	[45]
		Colombia	13.54	10.99	-	[99]
	Buckberry and Chamberlain, 2002 [12]	UK	9.67	10.56	-	[12]
		USA	13.39	12.38	-	[36]
		UK	-	-	9.8	[38]
		Spain	-	-	12.87	[35]
		Italy	11.8	13.9	-	[101]
		Spain	-	-	11.24	[47]
		Greece	11.72	11.18	-	[43]
		Thailand	14.5	15.4	-	[51]
		Greece	-	-	16.47	[44]
		Colombia	-	-	7.87	[56]
		Greece	14.2	13.3	-	[45]
		Colombia	12.5	12.17	-	[99]
	Osborne et al., 2004 [100]	USA	-	-	11.4	[100]
		Thailand	12.2	12.2	-	[51]
		USA (E)	-	-	15.61	[17]

### Future lines of research in age estimation

Researchers have been focusing on refining mathematically advanced methods to minimize the errors made in the different age estimation procedures (e.g., [26, 102–108]) or have ventured to create or renovate aging methodologies (e.g., [32, 34, 68, 100, 109–113]). Lately, much information has also been published about new updated approaches based on digital images, CT scanners, and 3D models [114–131], which, although they cannot fully replace the necessary documented dry-bone collections, have relevant advantages. In addition to being easily and cheaply stored, they could be a great solution to the loss of data in reburied collections and can be shared between institutions/researchers without the necessity of synchronous contact.

Thus, despite that this new technology has been recently applied, at least partially, to some of the acetabular-based estimation methodologies [62–64], some relevant microanatomical details such as different types of porosity, diverse textures, or color shades could not be displayed through CTs so far. Thus, the impossibility to examine the more subtle bony alterations at the 3D images of the auricular surface has already been reported [132]. As interesting it is, the detailed acetabular aging traits reported by Rissech et al. [15] and San-Millán et al. [34] hardly will be able to be assessed by computed tomography in its whole, making the dry bones and the skeletal collections irreplaceable. However, and despite these difficulties, using regression models based on Calce’s method [32] and some variables from SanMillán-Rissech’s method [34], mean absolute errors of 9.20 and 9.15 years, respectively, were reported using 250 and 400 Indian CTs, respectively [63, 64]. In addition, a complementary summary age model generated based on PCA procedures get a mean absolute error of 7.06 years, similar to the original results from San-Millán et al. [34]. Despite CTs procedures can be convenient and advantages to reduce the time-consuming process of maceration in some cases, standard tools are still meaningful and opportune for working with fully skeletonized remains, found periodically in forensic cases but as a daily basis in bioarcheology contexts. Thus, these collections are still fundamental to provide information and knowledge to researchers in order to enable a better comprehension of the poorly understood progression of skeletal senescence [133]. As documented collections continue to be amassed in Africa, Asia, and South America, they provide the perfect opportunity for continued research in inter-population differences outside of the standardized US and/or Eurocentric research [134–142].

## Conclusion

The existence of significant inter-population differences in the timing of acetabular aging has been proved between European North Americans and Portuguese, especially in females, but the multiple underlying factors determining these differences require further research. These differences in the rates of maturation need to be understood prior to the systematic application of the acetabular aging method, since they can play a significant role in the inaccuracy of the method’s results. Even though using population-specific reference samples seems to significantly refine acetabular-based age estimation methodologies as a global pattern and enhances its applicability reducing the invalid cases reported by the IDADE2 software, SanMillán-Rissech’s methodology results in lower inaccuracy values in comparison with other pelvic-based techniques (pubic symphysis and auricular surface) regardless of the geographical origin of the reference sample. Despite that population-specific reference samples are recommended, the acetabular traits still work well in many contexts globally. Thus, stronger outcomes and better preservation should place the acetabulum above other pelvic age markers often used regularly in forensic and bioarcheological contexts.

## Data Availability

N/A.
